# Adult Prg4^+^ progenitors repair long-term articular cartilage wounds in vivo

**DOI:** 10.1172/jci.insight.167858

**Published:** 2023-09-08

**Authors:** Mei Massengale, Justin L. Massengale, Catherine R. Benson, Ninib Baryawno, Toshihiko Oki, Matthew L. Steinhauser, Alissa Wang, Deepak Balani, Luke S. Oh, Mark A. Randolph, Thomas J. Gill, Henry M. Kronenberg, David T. Scadden

**Affiliations:** 1 Spaulding Rehabilitation Network at Mass General Brigham, Cambridge, Massachusetts, USA.; 2 Department of Medicine, Endocrine Unit, and; 3 Center for Regenerative Medicine, Massachusetts General Hospital, Boston, Massachusetts, USA.; 4 Harvard Stem Cell Institute, and; 5 Department of Stem Cell and Regenerative Biology, Harvard University, Cambridge, Massachusetts, USA.; 6 Department of Medicine, Rheumatology Unit, Massachusetts General Hospital, Boston, Massachusetts, USA.; 7 Department of Neurosurgery, Beth Israel Deaconess Medical Center, Boston, Massachusetts, USA.; 8 Aging Institute, University of Pittsburgh School of Medicine, Pittsburgh, Pennsylvania, USA.; 9 Department of Medicine, Division of Cardiovascular Medicine, Brigham and Women’s Hospital, Boston, Massachusetts, USA.; 10 Department of Orthopedic Surgery, and; 11 Department of Plastic Surgery, Massachusetts General Hospital, Boston, Massachusetts, USA.

**Keywords:** Cell Biology, Adult stem cells, Bone marrow, Cartilage

## Abstract

The identity and origin of the stem/progenitor cells for adult joint cartilage repair remain unknown, impeding therapeutic development. Simulating the common therapeutic modality for cartilage repair in humans, i.e., full-thickness microfracture joint surgery, we combined the mouse full-thickness injury model with lineage tracing and identified a distinct skeletal progenitor cell type enabling long-term (beyond 7 days after injury) articular cartilage repair in vivo. Deriving from a population with active Prg4 expression in adulthood while lacking aggrecan expression, these progenitors proliferate, differentiate to express aggrecan and type II collagen, and predominate in long-term articular cartilage wounds, where they represent the principal repair progenitors in situ under native repair conditions without cellular transplantation. They originate outside the adult bone marrow or superficial zone articular cartilage. These findings have implications for skeletal biology and regenerative medicine for joint injury repair.

## Introduction

Physical proximity of bone marrow and articular surfaces to sites of joint cartilage injury has led to the hypothesis that these tissues may contain progenitors for joint cartilage full-thickness repair (1–8). Early histomorphometry studies (1) suggested a bone marrow origin of these repair cells but were generally unable to discern lineage relationships. Fate mapping using constitutive Cre constructs (3, 4) showed that bone marrow and synovium might contain repair progenitors, but the broad labeling of diverse tissues (cartilage, synovium, and bone marrow) prior to injury limits the ability to discern the specific origin of the responding cells (Supplemental Figure 1A; supplemental material available online with this article; https://doi.org/10.1172/jci.insight.167858DS1). When combined with injury, constitutive Cre models may introduce additional ambiguity because the promotor that drives Cre itself might be induced by injury. Based on these fate-mapping studies alone (3, 4), it remains unclear whether the labeled wound cells originate from synovium, adult marrow, or articular cartilage.

Using transplantation (3, 4), donor cells from adult marrow or synovium were shown to be capable of engraftment into cartilage wounds. In particular, donor cells from synovium demonstrated mesenchymal stem cell (MSC) properties distinct from earlier reports (3, 9, 10), shifting the focus from marrow to synovium. Despite these advances (3, 4), the identity of progenitors under native repair conditions without exogenous cellular input remains unclear.

Work using nonconstitutive (inducible) Cre models showed that activated skeletal stem cells do not come from systemic delivery but from the local chondral surface (5). However, due to its broad expression in diverse tissues, the β-actin promoter used in that study lacks lineage specificity and limits the interpretation of the cell or tissue of origin (5). Using an inducible model (11), acute wounds at 7 days post injury (dpi) showed cells in wounds that were traceable to a shared origin of synovium and articular cartilage, but later time points beyond 7 dpi were not assessed (11). Despite extensive studies, the precise identity of the native progenitors for adult long-term repair of cartilage remains ambiguous (12).

To address these questions, we used inducible mouse models with a full-thickness injury model (13), which provides bone marrow stromal cells (BMSCs) open access to the cartilage wounds and simulates human microfracture surgery. A partial-thickness injury model was not selected in this context because it does not simulate repair induced by human microfracture surgery, the common surgery performed for cartilage regeneration. Long-term wounds were followed for 60 days after injury to identify native adult repair progenitors in the mouse and their tissue origin. We also used mRNA fluorescence in situ hybridization (FISH) assays to assess whether chondrocyte-specific genes were expressed in the labeled wound cells.

## Results

### Adult superficial zone chondrocytes do not reconstitute cartilage wounds.

**Figure 1:**
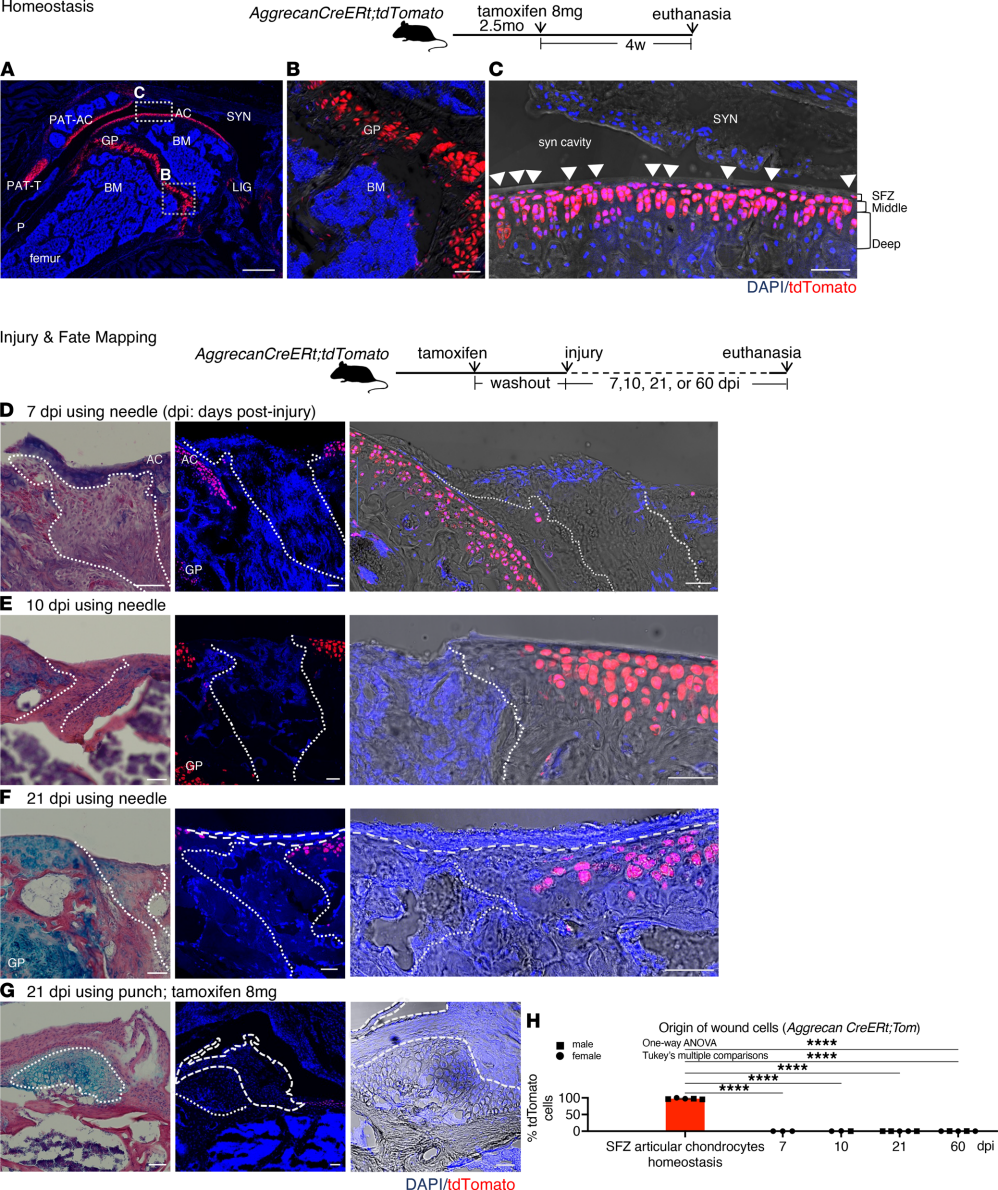
Adult aggrecan-lineage cells in homeostasis and articular cartilage wounds. (**A**–**C**) Homeostasis: Fluorescence images of *Aggrecan* *CreERt;tdTomato* mouse knee articular cartilage. (**A**) Overview. (**B**) Marrow. (**C**) Maximum intensity projection (projection of brightest signals only). AC, articular cartilage; GP, growth plate; BM, marrow; SYN, synovium; P, periosteum; LIG, ligaments; PAT-C, patellar cartilage; PAT-T, patellar tendon; JC, joint capsule. Dotted lines: tidemark (boundary between noncalcified and calcified cartilage). Arrowheads: superficial zone (SFZ) chondrocytes. (**D**–**G**) Injury: Alcian blue and fluorescence images (low and high magnification) of adjacent sections of cartilage wounds of *Aggrecan* *CreERt;tdTomato* at 7, 10, and 21 dpi (**D**–**G**), with 4 mg tamoxifen, 1.5-week washout allowing tamoxifen metabolism, and then injury using needle (**D**–**F**). (**G**) Tamoxifen (8 mg), 6-week washout allowing aggrecan-expressing marrow stromal cells to proliferate, and then injury using 0.3-mm-diameter biopsy punch. Dashed contours: soft tissue superficial to the wound. Dotted contours: wound boundary determined by histology, brightfield, or fluorescence images. Red, tdTomato. Blue, DAPI. Scale bar: 500 μm (**A**) and 50 μm (**B**–**G**). (**H**) Quantification: aggrecan-lineage cells in homeostasis vs. wounds. SFZ chondrocytes were defined as flat cells in the top 2 layers of articular chondrocytes superficial to the underlying middle and deep zones, excluding free cells outside the cartilage matrix or tissue without 3-zonal structure typical of articular cartilage (e.g., the periphery of articular cartilage or soft tissue attachment points). Wound excluded surrounding soft tissues. Also see Methods. Sample size: homeostasis, *n* = 5 (8 mg tamoxifen, *n* = 3; 10 mg, *n* = 2); 7 dpi, *n* = 3 (all using needle); 10 dpi, *n* = 3 (all needle, with 1 receiving 8 mg tamoxifen); 21 dpi, *n* = 5 (needle, *n* = 2; punch, *n* = 3), with 1 in punch group receiving 8 mg tamoxifen; 60 dpi, *n* = 5 (all punch; 2 were aged mice in Supplemental Figure 3F). One-way ANOVA, F = 11737 (degrees of freedom between groups = 4 and within groups = 16). ****P < 0.0001.

Articular cartilage has been hypothesized to contain progenitors for full-thickness injury repair. Particularly, the superficial zone (SFZ) chondrocytes, defined as the flat cells in the top 2 layers of articular chondrocytes superficial to underlying middle and deep zones (Figure 1, A–C), have been demonstrated to be progenitors for postnatal growth (14). To test whether these cells make cellular contributions to adult repair, we selected a model that marks articular chondrocytes: *Agc1^tm(IRESCreERT2)^*;*Gt(ROSA)26Sor^tm9(CAG-tdTomato)Hze^* mice (referred to as *Aggrecan CreERt;tdTomato* mice), a tamoxifen-inducible, Cre-lox construct driven by the promoter of the *ACAN* gene encoding the major cartilage protein, aggrecan (15). Tamoxifen (8 mg) administration, given to 2.5-month-old *Aggrecan CreERt;tdTomato* mice, led to labeling of a small portion of BMSCs and a portion of the patella tendon, and the majority of growth plate and articular chondrocytes (Figure 1, A–C), particularly nearly all of the adult SFZ chondrocytes (Figure 1, A–C, and H).

To assess whether these tdTomato^+^ cells representing aggrecan-lineage cells in homeostasis reconstitute the wound after injury*, Aggrecan CreERt;tdTomato* mice were administered 4 mg tamoxifen, followed by a 1.5-week “washout” to allow tamoxifen metabolism and elimination, and then full-thickness injury of the right knee. Wounds evaluated showed rare or no tdTomato^+^ cells at 7, 10, and 21 dpi (Figure 1, D–F). To verify this, we adopted an orthogonal injury technique, using 0.3-mm tissue biopsy punches instead of needles to generate the full-thickness wounds (Supplemental Figure 1, B and C). We also varied experimental conditions by doubling the tamoxifen dose to 8 mg (Figure 1G and Supplemental Figure 2). Finally, we evaluated longer-term wounds at 60 dpi and tested older animals (11.5 months of age; Supplemental Figure 3). Despite these variations, the results confirmed rare or no tdTomato^+^ cells (aggrecan-lineage cells) in the wounds at all time points (Figure 1H). We interpreted this to mean that adult aggrecan lineages in homeostasis, representing most adult articular chondrocytes, do not make cellular contributions to the full-thickness adult cartilage wound.

### Adult Prg4-lineage cells reconstitute long-term cartilage wounds.

**Figure 2:**
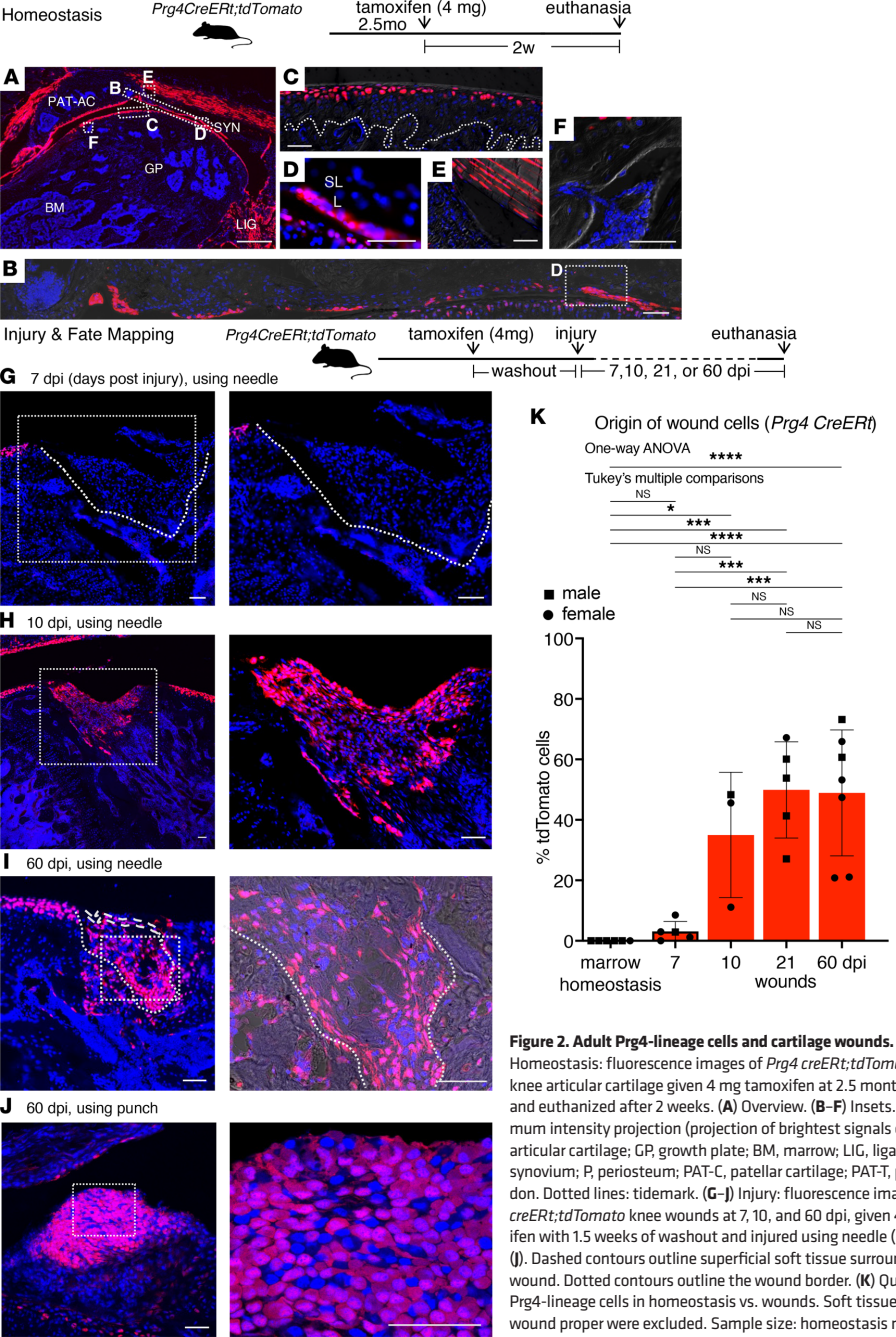
Adult Prg4-lineage cells and cartilage wounds. (**A**–**F**) Homeostasis: fluorescence images of *Prg4 creERt;tdTomato* mouse knee articular cartilage given 4 mg tamoxifen at 2.5 months of age and euthanized after 2 weeks. (**A**) Overview. (**B**–**F**) Insets. (**B**) Maximum intensity projection (projection of brightest signals only). AC, articular cartilage; GP, growth plate; BM, marrow; LIG, ligaments; SYN, synovium; P, periosteum; PAT-C, patellar cartilage; PAT-T, patellar tendon. Dotted lines: tidemark. (**G**–**J**) Injury: fluorescence images of *Prg4 creERt;tdTomato* knee wounds at 7, 10, and 60 dpi, given 4 mg tamoxifen with 1.5 weeks of washout and injured using needle (**G**–**I**) or punch (**J**). Dashed contours outline superficial soft tissue surrounding the wound. Dotted contours outline the wound border. (**K**) Quantification: Prg4-lineage cells in homeostasis vs. wounds. Soft tissues outside the wound proper were excluded. Sample size: homeostasis marrow (*n* = 6); 7 dpi (*n* = 5, 2 received 6 mg tamoxifen, all using needle); 10 dpi (*n* = 3, needle); 21 dpi (*n* = 5, 4 needle with 2 using 27G, and 1 punch); 60 dpi (*n* = 7, 4 needle with 2 using 27G, and 3 punch). One-way ANOVA, F = 15.57 (degrees of freedom between groups = 4 and within groups = 21). *P < 0.05; ***P < 0.001; ****P < 0.0001.

To clarify the identity of wound-reconstituting cells, we next evaluated similarly aged *Prg4^tm1(GFP/cre/ERT2)Abl/J^;Gt(ROSA)26Sor^tm9(CAG-tdTomato)Hze^* mice (referred to as *Prg4 CreERt;tdTomato* mice) (14, 15) driven by the promotor/enhancer region of the *Prg4* gene encoding the joint lubricating protein, proteoglycan4 or lubricin (14). Tamoxifen (4 mg) led to tdTomato labeling of SFZ chondrocytes and diverse soft tissues both inside and outside the joint cavity (intraarticular and extraarticular): the synovium extending to the intra-articular portion of periosteum, contiguous with the extra-articular portion of the periosteum; the tenosynovium, the extra-articular synovium covering of the tendons, also known as tendon sheath; ligaments; entheses; and the joint capsule, but not in growth plate or bone marrow (Figure 2, A–F; *n* = 6). Increasing the tamoxifen dose to 12 mg did not lead to detectable tdTomato^+^ cells in the growth plate or bone marrow (Supplemental Figure 4; *n* = 4).

Using 4 mg tamoxifen and the same needle-induced full-thickness injury, tdTomato^+^ cells in the wounds of *Prg4 CreERt;tdTomato* mice were rare or absent at 7 dpi, but became abundant at 10, 21, and 60 dpi (Figure 2, G–K), with wound architecture shown in Supplemental Figure 5. We also verified the results using a smaller 27G needle (0.4 mm width) instead of the typical needle size of 25G (0.5 mm width), with such wounds at 21 dpi (*n* = 2) and 60 dpi (*n* = 2) showing abundant tdTomato^+^ cells as well. Wound tdTomato^+^ cells were unlikely, due to native cartilage being “pushed” into the wounds given the lack of labeled cells in the aggrecan model (Figure 1, D–H). To further validate our results, we used the orthogonal biopsy punch injury technique described above (Supplemental Figure 1C). Tissue removal was verified by the inspection of the tissue ejected by the plunger of the biopsy punch, or inspection of the surgical wound with a table-mounted surgical magnifier or operating microscope. Using the biopsy punch, tdTomato^+^ cells in wounds remained abundant at 60 dpi (Figure 2K), while they were absent in vehicle controls (Supplemental Figure 6), consistent with the data acquired using the conventional needle approach. These data, combined and summarized in Figure 2K, indicate that adult Prg4-lineage cells found in surrounding tissues outside the bone marrow are cells that reconstitute the cartilage wounds even long after injury.

**Figure 3:**
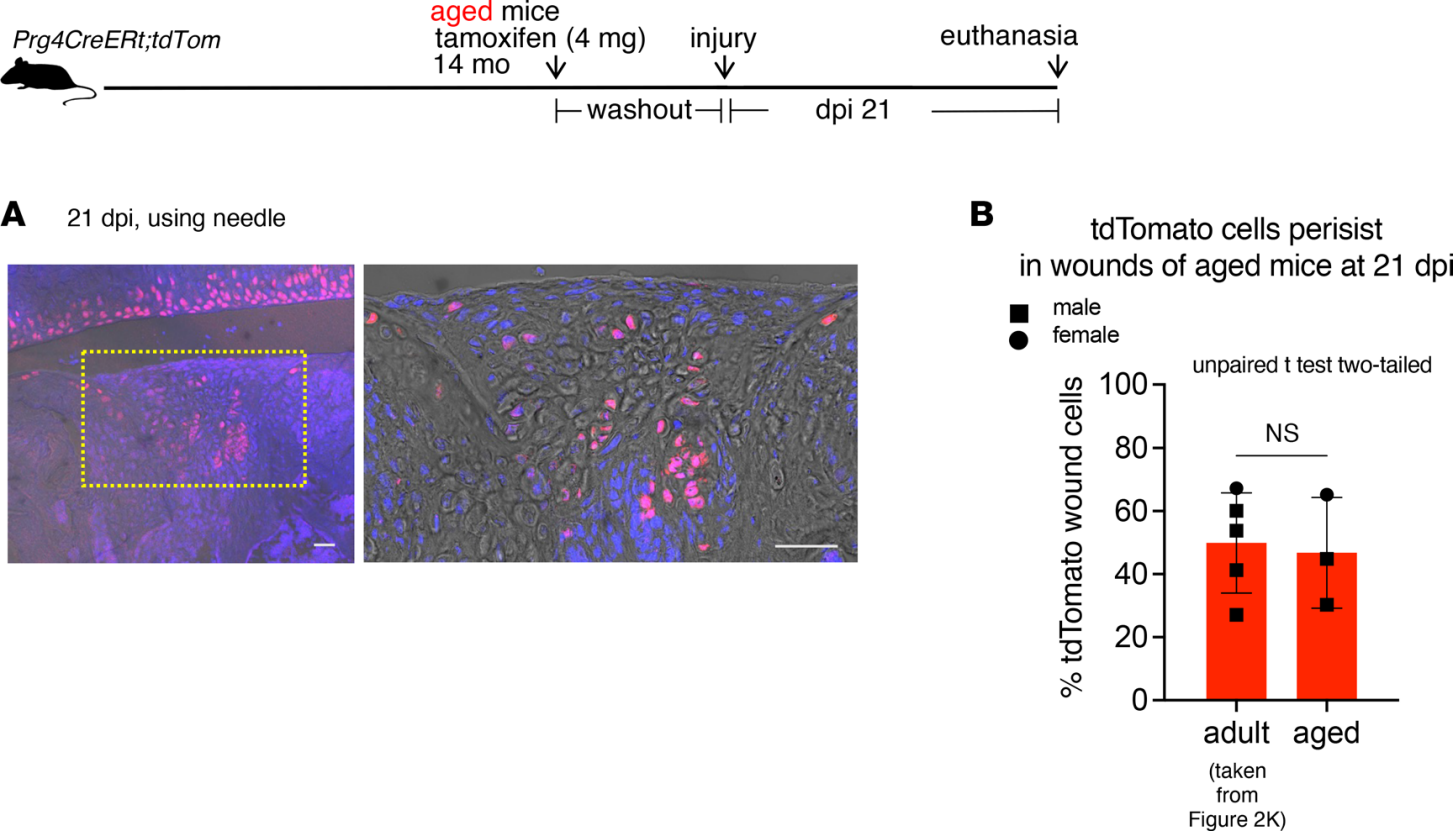
Adult Prg4-lineage cells in the wounds of adult and aged mice. (**A**) Injury of aged *Prg4 creERt;tdTomato* mice: fluorescence images of wound at 21 dpi with 4 mg tamoxifen given at 14 months of age and injured with needle. Red: tdTomato. Blue: DAPI. Scale bar: 50 μm. (**B**) Quantification: Prg4-lineage cells in knee wounds at 21 dpi, adult vs. aged mice. Superficial soft tissues outside the wound proper were excluded. Sample size: *n* = 5 from Figure 2K, and *n* = 3 for aged mice (14–15 months), all using needle. Unpaired *t* test, 2-tailed, *t* = 0.2605, degrees of freedom = 6, *P* = 0.8032.

To determine whether adult Prg4-lineage cells populate cartilage wounds at a more advanced age, we gave tamoxifen to aged mice followed by the injury and observed that abundant tdTomato^+^ cells populated the wounds at 14 dpi (Supplemental Figure 7A) and 21 dpi (Figure 3, A and B), with wound architecture shown in Supplemental Figure 7, B–E.

### Adult Prg4-lineage cells are adult cartilage repair progenitors in vivo.

**Figure 4:**
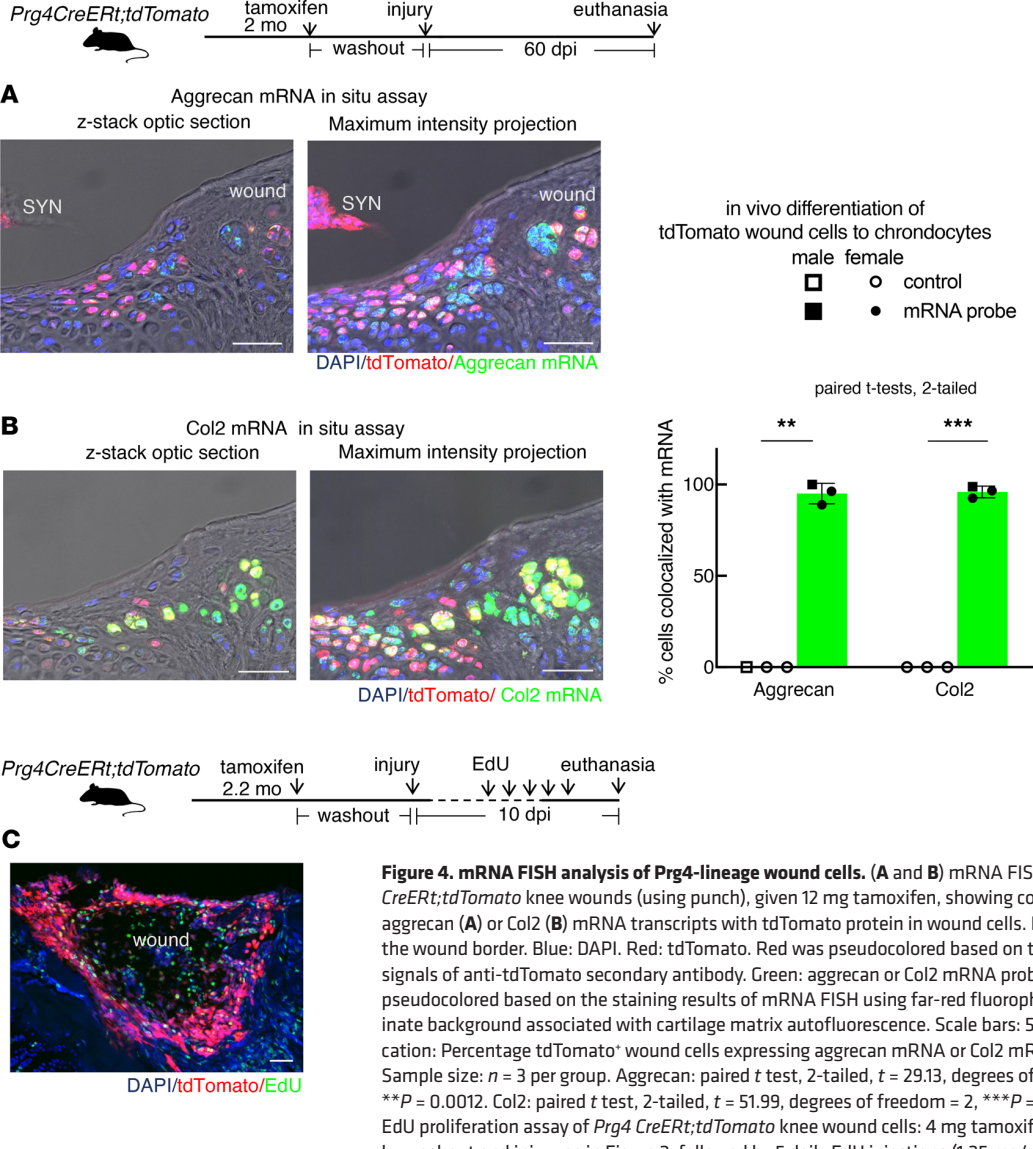
mRNA FISH analysis of Prg4-lineage wound cells. (**A** and **B**) mRNA FISH assay of *Prg4* *CreERt;tdTomato* knee wounds (using punch), given 12 mg tamoxifen, showing colocalization of aggrecan (**A**) or Col2 (**B**) mRNA transcripts with tdTomato protein in wound cells. Dotted lines: the wound border. Blue: DAPI. Red: tdTomato. Red was pseudocolored based on the staining signals of anti-tdTomato secondary antibody. Green: aggrecan or Col2 mRNA probe. Green was pseudocolored based on the staining results of mRNA FISH using far-red fluorophores to eliminate background associated with cartilage matrix autofluorescence. Scale bars: 50 μm. Quantification: Percentage tdTomato^+^ wound cells expressing aggrecan mRNA or Col2 mRNA transcripts. Sample size: *n* = 3 per group. Aggrecan: paired *t* test, 2-tailed, *t* = 29.13, degrees of freedom = 2, **P = 0.0012. Col2: paired *t* test, 2-tailed, *t* = 51.99, degrees of freedom = 2, ***P = 0.0004. (**C**) EdU proliferation assay of *Prg4* *CreERt;tdTomato* knee wound cells: 4 mg tamoxifen followed by washout and injury as in Figure 2, followed by 5 daily EdU injections (1.25 mg/animal) prior to euthanasia at 10 dpi, and the last dose within 48 hours of euthanasia. Inset: maximum intensity projection (projection of brightest signals only). Red: tomato. Blue: DAPI. Green: EdU. Green was pseudocolored based on the staining signals of far-red fluorophores to eliminate the background associated with cartilage matrix autofluorescence. Scale bars: 50 μm.

At 60 dpi, the long-term wound matrix demonstrated Alcian blue positivity, providing evidence for matrix healing in all 7 animals analyzed: 5 with tamoxifen injection (Figure 2K and Supplemental Figure 5) and 2 vehicle controls (Supplemental Figure 6). To characterize the Prg4-lineage cells in the long-term (60 dpi) wounds, we performed mRNA FISH and showed they expressed mRNA transcripts of aggrecan (98.1%, *n* = 1 given 4 mg tamoxifen; 95.1% ± 5.7%, *n* = 3 given 12 mg tamoxifen) and type II collagen (100%, *n* = 1 given 4 mg tamoxifen; 96.0% ± 3.2%, *n* = 3 given 12 mg tamoxifen) (Figure 4, A and B), compared with controls (Supplemental Figure 8). Prg4-lineage wound cells were also found to proliferate, incorporating 5-ethynyl-2′-deoxyuridine (EdU) (Figure 4C). These results indicate that Prg4-lineage cells populate the wounds and differentiate into chondrocytes in vivo, distinguishing them from fibrous scar-forming cells. They serve as adult articular cartilage progenitors for repair in vivo.

### Adult Prg4-lineage progenitors predominate in wounds, do not originate in pre- or postinjury bone marrow.

**Figure 5:**
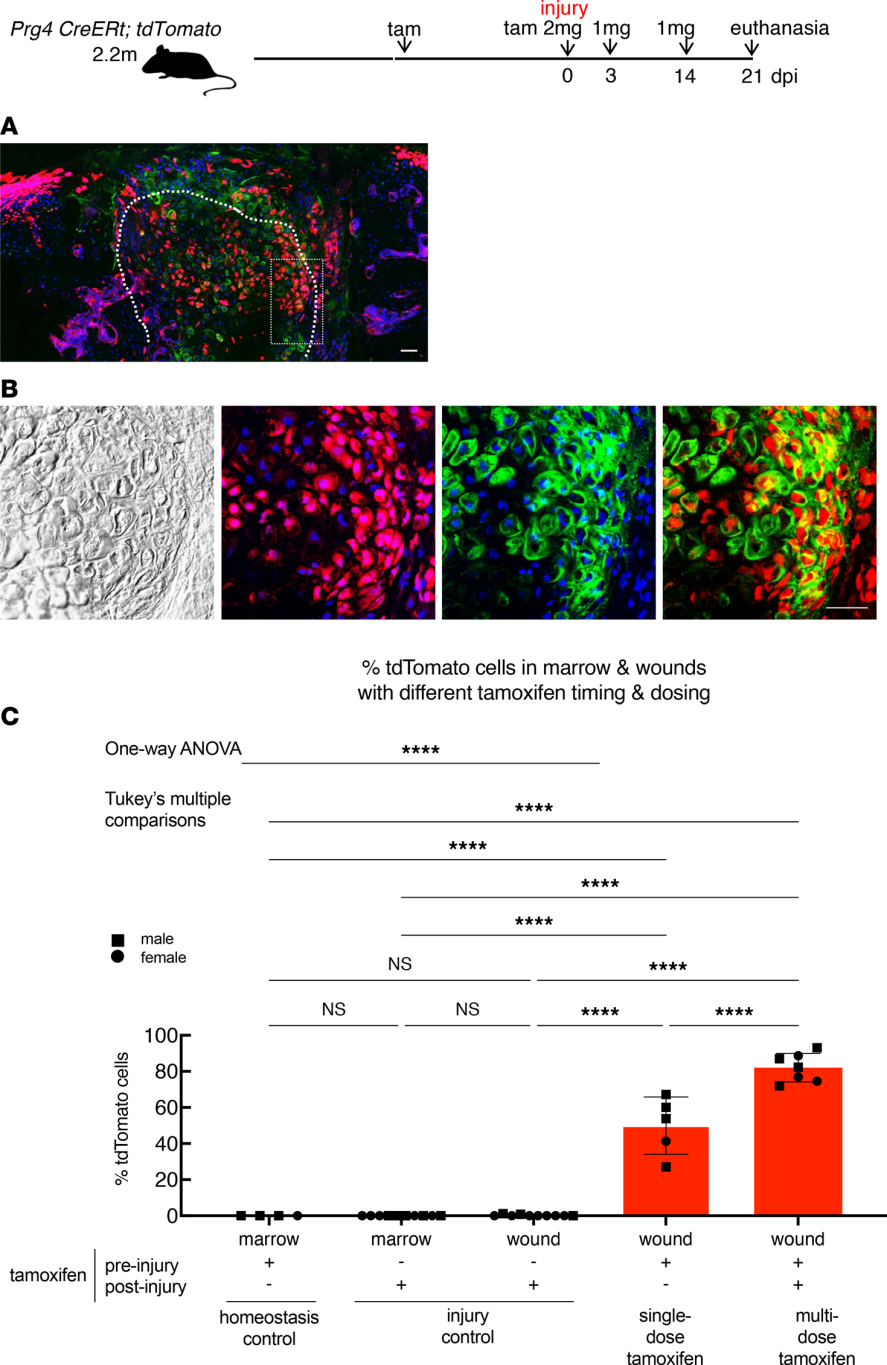
Abundance of adult Prg4-lineage progenitors in pre- and postinjury bone marrow and cartilage wounds. (**A** and **B**) Confocal fluorescence images with maximum intensity projection of *Prg4* *CreERt;tdTomato* knee wound (using punch) at 21 dpi, having received 6 mg tamoxifen before injury and 4 mg after injury (2 mg at injury, 1 mg at 3 dpi, and 1 mg at 14 dpi). Dotted lines: the wound border. Repaired cartilage matrix in aggrecan protein immunofluorescent staining (green). Green was pseudocolored based on staining signals of far-red fluorophores conjugated to secondary antibodies to eliminate the background associated with cartilage matrix autofluorescence. Scale bars: 50 μm. (**C**) Quantification: Percentage tdTomato^+^ cells in marrow and wounds with different tamoxifen timing and dosing: marrow homeostasis control (12 mg tamoxifen before injury with data taken from experiments related to Supplemental Figure 4 as described in the text), marrow and wound injury control (injury first, then tamoxifen, reversing the sequence of tamoxifen and injury protocol used in Figure 2, and euthanasia within 2–4 days to detect active Prg4-expressing cells in postinjury marrow and wounds, with data taken from Supplemental Figure 9). Single-dose tamoxifen: 4 mg preinjury only (data taken from Figure 2K); multiple-dose tamoxifen: before injury (6 mg *n* = 3 or 12 mg *n* = 4) and after injury (all 4 mg). Sample size: homeostasis control marrow (12 mg tamoxifen, *n* = 4, data taken from experiments related to Supplemental Figure 4 as described in the text); injury control marrow and wounds (*n* = 10 combining all time points from Supplemental Figure 9); single-dose tamoxifen wounds (*n* = 5 from Figure 2K); multiple-dose tamoxifen wounds (total *n* = 7). One-way ANOVA, F = 227.0 (degrees of freedom between groups = 4 and within groups = 31). ****P < 0.0001.

Since our observations thus far did not account for 100% of the wound cells, we gave multiple doses of tamoxifen before and after injury and found that tdTomato^+^ wound populations became the predominant wound cell population at 21 dpi (82.1% ± 7.9%, *n* = 7; Figure 5, A–C). While demonstrating no signals in negative controls, these cells, as well as the cartilage matrix, showed positive immunoreactivity using an anti-aggrecan antibody (Figure 5, A and B), providing evidence for matrix healing. In wound cells, tdTomato and aggrecan protein overlapped in the cytoplasm but had distinct spatial patterns, as expected for a nuclear and cytoplasmic protein such as tdTomato versus a protein in the secretory pathway such as aggrecan.

Because giving tamoxifen after injury might have marked cells directly if injury itself induced active Prg4 expression in wound cells, we tested this possibility by injuring *Prg4 CreERt;tdTomato* mice first and then giving tamoxifen (reversing the sequence of tamoxifen and injury used in Figure 2), as depicted in Supplemental Figure 9. This timing of tamoxifen labeled no or extremely rare cells in postinjury bone marrow or wounds at all time points assessed (up to 20 dpi), while tissues outside the wound such as articular chondrocytes and synovium were labeled as expected (Supplemental Figure 9). Together, these data validate the lineage tracing results from the multiple-dose tamoxifen experiments (Figure 5, A–C) and indicate that the tdTomato^+^ cells observed in these wounds were not the result of tdTomato labeling of cells actively expressing Prg4 in the wounds. Rather, these tdTomato^+^ cells in the wounds became tdTomato^+^ prior to entering the wounds; furthermore, they did not originate from either preinjury or postinjury bone marrow.

While these data do not establish the exact causes of the wide variability observed in this single-time-point tamoxifen treatment experiment (Figure 2K), such wide variability is dramatically reduced with multiple-time-point tamoxifen labeling (Figure 5C). Together, this raises the possibility that while single-time-point tamoxifen labeling (Figure 2) partially labels the progenitor pool, a broader tamoxifen labeling strategy (Figure 5) labels a larger progenitor pool. It is possible that additional adult Prg4^+^ progenitors continue to emerge after the initial single-time-point tamoxifen exposure, including time points after injury (11, 16). Such partial or underlabeling of the progenitor pool likely explains the wide variability in single-time-point tamoxifen-induced labeling (Figure 2K). Biological variability in tissue response to injury and the kinetics of healing likely plays a significant role.

### Adult-marrow-dwelling stromal cells (perinatal Sp7 lineage) are not major contributors to cartilage wound repair.

The absence of Prg4-lineage progenitors in pre- and postinjury bone marrow does not support adult marrow being the primary reservoir that supplies progenitors for adult full-thickness-injury repair. To directly test this question, we marked adult-marrow-dwelling stromal cells using an inducible mouse model, *Tg(Sp7-cre/ERT)1Hmk/0*;*Gt(ROSA)26Sor^tm9(CAG-tdTomato)Hze^*, referred to as *Sp7 CreERt;tdTomato*, driven by the transcription factor Sp7 (17). Previous work demonstrated that the developmental origin of a substantial portion of adult marrow stromal cells is traceable to a population that expresses Sp7 perinatally as early as E18.5 (17). In addition, this population responds to long bone fracture and differentiates to chondrocytes that participate in wound healing in vivo (17). We gave tamoxifen at peri- and postnatal time points and found abundant tdTomato^+^ cells in the adult marrow, consistent with a prior report (17), but rare or no tdTomato^+^ cells in articular cartilage wounds at 14 or 21 dpi (Supplemental Figure 10). Together, these data argue against adult marrow stromal cells as a primary cellular contributor to adult articular cartilage repair.

### Prg4^+^ articular chondrocytes, including those in the SFZ, are unlikely contributors to wound repair.

**Figure 6:**
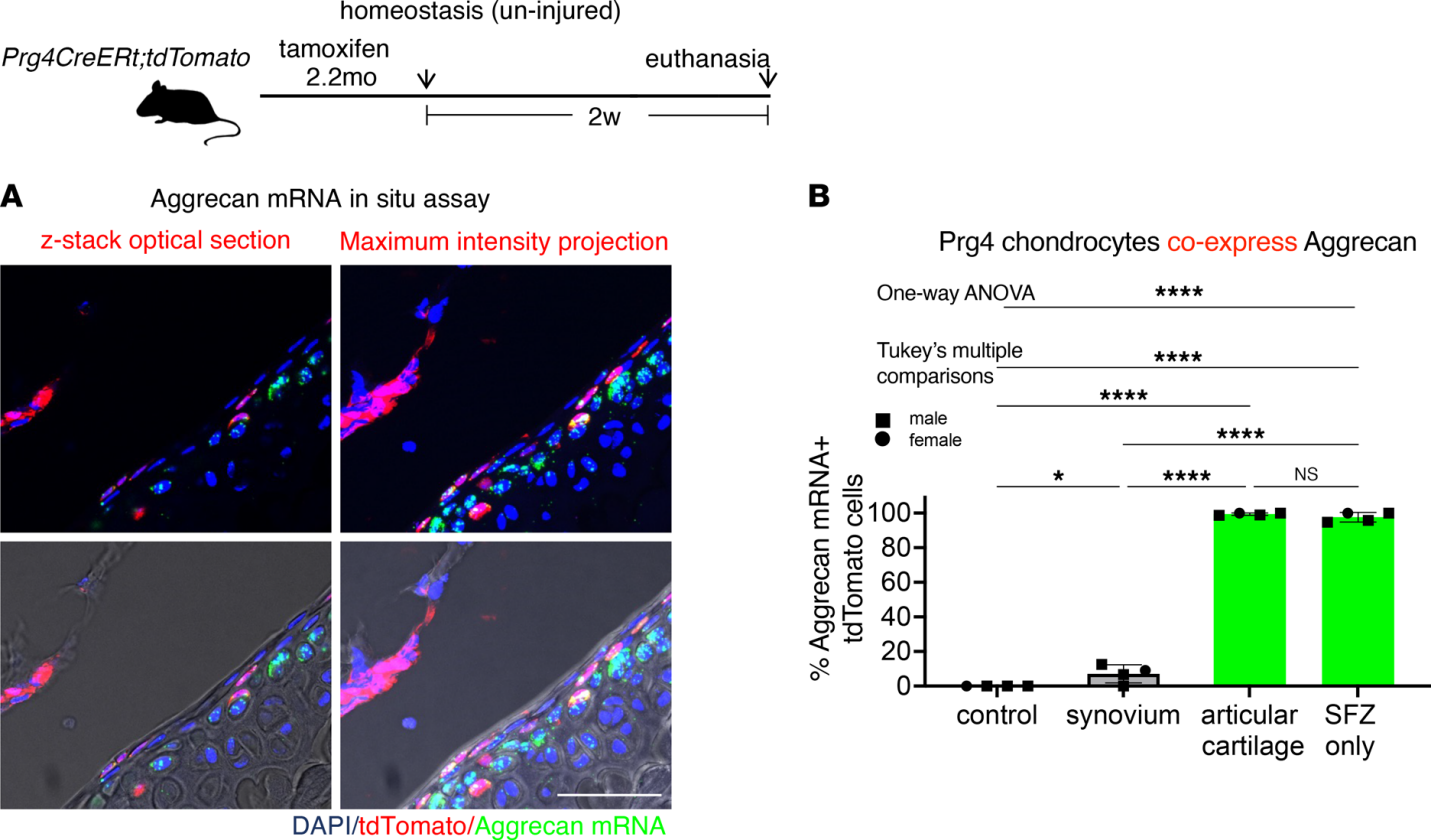
mRNA FISH analysis of Prg4^+^ articular chondrocytes. (**A**) Confocal fluorescence images of aggrecan mRNA FISH assay in *Prg4 CreERt2;tdTomato* knee articular chondrocytes with mice following the protocol used in Figure 2A, with aggrecan mRNA probe showing articular chondrocytes: single *Z* section and maximum intensity projection (projection of maximum intensity signals only). The *z*-axis thickness within 16 μm was selected to be smaller than 20 μm, the typical diameter of a chondrocyte, to avoid positional artifacts in the maximum intensity projection. Blue: DAPI. Red: tdTomato. Red was pseudocolored based on the staining signals of anti-tdTomato secondary antibody. Green: aggrecan mRNA transcripts. Green was pseudocolored based on FISH staining using far-red fluorophores to eliminate the background associated with cartilage matrix autofluorescence. Scale bars: 50 μm. (**B**) Quantification: Percentage Prg4^+^ (tdTomato^+^) cells in synovium, articular cartilage, and SFZ that express aggrecan mRNA (*n* = 4); SFZ chondrocytes were defined as flat cells in the top 2 layers of articular chondrocytes, superficial to the underlying middle and deep zones, excluding free cells outside the cartilage matrix or tissue without zonal architecture typical of articular cartilage, e.g., the periphery of articular cartilage or soft tissue attachment points. One-way ANOVA: F = 1336 (degrees of freedom between groups = 3 and within groups = 12). *P < 0.05; ****P < 0.0001.

Since approximately 30% of articular chondrocytes, of which SFZ chondrocytes are a subset, express tdTomato protein in tamoxifen-exposed *Prg4* *CreERt;tdTomato* mice (Figure 2C), we assessed whether these Prg4-lineage articular chondrocytes can populate cartilage wounds. Using mRNA FISH, we found that 97.6% ± 2.8% (*n* = 4) of Prg4^+^ SFZ chondrocytes and 99.4% ± 0.7% (*n* = 4) of all Prg4^+^ articular chondrocytes coexpress aggrecan (Figure 6; control images in Supplemental Figure 11). Since aggrecan CreER–expressing cells fail to participate in wound repair (Figure 1, D–H), the SFZ cells expressing Prg4 are unlikely to serve as progenitors in injury repair. Therefore, we conclude that the repair cells come from Prg4^+^ cells outside the articular cartilage.

## Discussion

These data identify adult Prg4-expressing cells as progenitors for adult articular cartilage injury repair. These cells proliferate and differentiate to become chondrocytes that express genes such as *ACAN* and *Col2*. Historically, it has been hypothesized that cells involved in cartilage repair primarily come from bone marrow, the chondral surface, or synovium. Supportive studies have often used constitutive Cre models that limit the ability to determine when the reporter gene is expressed in development or in relation to injury. We used inducible and distinct reporter gene models to overcome some of those limitations. In so doing, our data argue against bone marrow or chondral surface cells as the primary cell reservoir for native repair (although we cannot rule out some contribution). Rather, periarticular soft tissue is the likely source, with further studies being necessary to define the role of synovium or other soft tissue types. Synovium lines the synovial joint cavity and associated structures such as ligaments and tendon. An expanded definition of synovium lining also includes the wrapping of tendon and ligaments and lining of bursae in periarticular soft tissue (18), given terms such as tenosynovium or tendon sheath. Deeper in the lining layer is the sublining layer, continuous with fat and other connective tissue. Since adult Prg4^+^ cells mostly are present in the lining layer, the periarticular synovial lining layer likely is a reservoir for progenitors for cartilage repair.

Since Prg4^+^ cells are also seen in the meniscus prior to injury (14), although meniscal chondrocytes express aggrecan (15), we cannot exclude the possibility that some cells in the meniscus might contribute to articular cartilage repair. However, this is beyond the scope of this study, and future studies are needed to determine this.

Our conclusion is supported by recent transcriptome analysis suggesting that lining Prg4^+^ cells undergo hyperplasia after cartilage injury, although cartilage wounds were not examined, and time points examined were limited to 6 dpi in that study (16). Trajectory analysis in another study (19) predicted that Dpp4^+^ mesenchymal progenitors in fat, a part of the mesenchymal interstitium (20), give rise to Prg4^+^ lining cells. If this is the case in vivo, the fat may serve to replenish the Prg4-progenitor pool in the setting of injury. Future studies will be needed to investigate this in vivo.

We did not find evidence that the Prg4-lineage cells arrived via vasculature at the wound bed. Examination of adult homeostatic marrow, which is rich in blood vessels, did not reveal any Prg4-lineage cells (Figure 2F and Supplemental Figure 4; *n* = 10 total with 6 given 4 mg tamoxifen and 4 given 12 mg tamoxifen). Also, no or rare Prg4-lineage wound cells were observed during the acute time points after injury (≤7 dpi) when vascular disruption occurs. These findings are not definitive but consistent with a prior report by Murphy et al. showing that progenitors are not from the systemic blood circulation, as evaluated by parabiosis (5).

Similarly, we do not consider it likely that our analysis was confounded by tdTomato^+^ cells potentially being “pushed into” the wounds. Such contamination would likely affect all Cre models used. The consistent rarity or absence of tdTomato^+^ cells in wounds at all time points from all mouse models — *Aggrecan* *CreERt;tdTomato*, *Sp7* *CreERt;tdTomato*, and *Prg4* *CreERt;tdTomato* at acute time points (≤7 dpi), with the exception of *Prg4* *CreERt;tdTomato* at later time points — together argue against such contamination being a significant confounder. Lastly, if rare Prg4-lineage SFZ chondrocytes were to be retained in the wounds as contaminants, the vast majority of such potential contaminants would be expected to coexpress aggrecan, a population that we defined as not contributing to repair. Aggrecan^–^Prg4^+^ SFZ cells would be a rare population within the small population of SFZ contaminants, and unlikely to be the major contributor to wound repair.

Sex-based differences in joint diseases have been observed both in clinical practice and scientific literature. Although not all groups had equal numbers of male and female mice, there is representation of males and females in all groups in Figures 1–6, except the *Aggrecan CreERt;tdTomato* dpi 7 group in Figure 1, and 1 homeostasis control group for *Sp7 CreERt;tdTomato* in Supplemental Figure 10. There were both male and females combining all time points tested in Supplemental Figure 9. Comparing the 2 sexes within each group did not reveal any distinctive trends. We focused on addressing the foundational question regarding the identity of adult native progenitors and found that this population serves in cartilage healing for both males and females. Although the sex-based difference is not the primary object of this study, these data can serve as a beginning point for future investigations on potential sex-based difference in articular cartilage healing.

The complex spatial in vivo data excluded the use of automatic imaging analysis tools, led to the intensity of microscopic analysis, and contributed to the limited mouse numbers in some groups. Despite this, given the effect size observed, the sample size sufficiently powers the statistical tests used in evaluating the hypotheses set forth.

These data have several other limitations. Not all wound cells were accounted for, possibly due to (a) Cre inefficiency, (b) the depletion or exhaustion of the tdTomato^+^ population labeled by the single tamoxifen dose, or (c) the continuous emergence of new cells that begin to express Prg4 for the first time that could not be captured as tdTomato^+^ cells without continuous administration of tamoxifen. Other minor populations may contribute to the wound that we did not detect using the Cre-lox approach. To account for 100% of the wound-cell lineages, additional sorting and sequencing of the wound cells would be needed. Furthermore, our data focused on in vivo repair progenitors using a full-thickness injury model and did not rule out the possibility that other populations could contribute to repair under other experimental conditions, e.g., partial-thickness repair or transplantation of marrow cells especially after ex vivo genetic manipulation, or other minor populations contributing to healing. Finally, this study examines the cells used for normal or native cartilage repair; this analysis does not preclude the possibility that cells such as bone marrow cells might be useful for repair clinically.

Our data do not support the historical paradigm of native cartilage repair progenitors for full-thickness injury coming from adult marrow or articular cartilage. This may contribute to the limitations of widely practiced procedures implanting BMSCs in cartilage wounds or joint cavities. Future studies will be required to determine whether these findings can help improve adult joint repair.

## Methods

### Blinding

Blinding was not possible for injury models due to the need for colony maintenance and was not possible for fluorescence image analysis due to the distinct pattern of tissue labeling for mouse models.

### Mice

Animal colony maintenance and experiments followed approved IACUC guidelines at Massachusetts General Hospital. The *Agc1^tm(IRESCreERT2)^*;*Gt(ROSA)26Sor^tm9(CAG-tdTomato)Hze^* mouse strain was gifted to us by Benoit de Crombrugghe (Department of Molecular Genetics, University of Texas MD Anderson Cancer Center, Houston, Texas, USA) and Yefu Li (Department of Developmental Biology, Harvard School of Dental Medicine, Boston, Massachusetts, USA), and Prg4^tm1(GFP/cre/ERT2)Abl/J^;*Gt(ROSA)26Sor^tm9(CAG-tdTomato)Hze^* strain was a gift from Andrew Lassar (Department of Biological Chemistry and Molecular Pharmacology, Harvard Medical School, Boston, Massachusetts, USA) and Matthew Warman (Boston Children’s Hospital, Boston, Massachusetts, USA). The *Tg(Sp7-cre/ERT)1Hmk/0*;*Gt(ROSA)26Sor^tm9(CAG-tdTomato)Hze^* mouse strain was a gift from Tatsuya Kobayashi (Endocrine Division, Massachusetts General Hospital, Boston, Massachusetts, USA). All mice were bred with Rosa tdTomato (C57BL6) females initially and maintained in mixed background, including S129 and CD1, in SPF rooms of the animal facility of Massachusetts General Hospital. Mice were identified by ear tags. Genotyping procedures followed a standard PCR-based genotyping protocol. Mice were euthanized using CO_2_ per IACUC protocol approved for this study. For experiments related to Figure 1, except those indicated in aging experiments, mice of estimated 2–5 months of age were used for homeostasis and injury models; for experiments related to Figures 2–6, mice 2–3 months of age were used, except as indicated in the aging experiments; for experiments related to Supplemental Figures, mice were 2–4 months of age, except as indicated in the aging experiments. In experiments related to Figure 1, a total of 16 mice (combining all postinjury time points) were examined. In experiments related to Figure 2, a total of 20 mice (combining all postinjury time points) were examined. In experiments related to Figure 3 and Supplemental Figure 7, a total of 4 aged mice (combining all postinjury time points 14 dpi, *n* = 1; 21 dpi, *n* = 3) were examined. In experiments related to Figure 4, A and B, a total of 4 mice were examined and in Figure 4C, a total of 3 mice were examined (10 dpi *n* = 1, 21 dpi *n* = 2). In experiments related to Figure 5, wound matrix aggrecan immunostaining of 2 mice (21 dpi *n* = 1, 60 dpi *n* = 1) was examined. In experiments related to Figure 6, a total of 4 animals were examined. In experiments related to Supplemental Figure 4, total of 4 mice were examined. In experiments related to Supplemental Figure 9, a total of 10 mice (combining all postinjury time points) were examined. In experiments related to Supplemental Figure 10, a total of 5 mice in homeostasis and 8 mice after injury (14 dpi *n* = 4 and 21 dpi *n* = 4) were examined. In experiments related to Supplemental Figure 11, a total of 4 animals were examined.

### Injury models

The full-thickness injury was performed as described in Fitzgerald et al. (13). After general anesthesia using a standard protocol of isoflurane and preoperative preparation and confirming absence of pain response, approximately 8-mm longitudinal skin wounds were made medial to the knee joint, followed by reflection of patella cartilage and ligament to expose the femur cartilage surface. A hypodermic needle (typically BD PrecisionGlide 25G needle, 305122) was positioned perpendicular to the chondral surface in the center region of the femoral condyle and advanced in a circular motion until tactile loss of resistance was achieved, indicating the complete fracture of subchondral bone. The fracture of subchondral bone was also confirmed further with the presence of blood at the wound upon withdrawal of the needle. Buprenorphine (0.1 mg/kg) was administered once prior to anesthesia and a total of 6 doses within 72 hours perioperatively for pain relief.

To offer an orthogonal injury model for the above and to validate that the results using the conventional needle approach were not due to “retained” tissue, we used 0.3-mm tissue biopsy punches (Shoney Scientific Inc., BPP BIPU17845 00556) that can extrude the removed tissue. The biopsy punch was positioned and advanced through the tissue in a similar fashion to what was used in the needle approach (13). Depth confirmation by tactile loss of resistance and the presence of blood upon withdrawal of the biopsy punch was achieved using the same technique described above. In addition, a hypodermic needle (27G) was inserted into the wound tract after biopsy punch injury to ensure the wound penetrated the subchondral bone. Tissue removal was verified by the inspection of the tissue ejected by the plunger of the biopsy punch, or inspection of the surgical wound with a table-mounted surgical magnifier or operating microscope. Buprenorphine (0.1 mg/kg) was administered once prior to anesthesia and a total 6 doses within 72 hours perioperatively for pain relief.

### Tamoxifen injection

Mice were injected with tamoxifen (Sigma-Aldrich, T5648) that had been dissolved at 10 mg/mL in corn oil (Sigma-Aldrich, C8267) in a 60°C water bath, aliquoted into Eppendorf tubes, and stored at –80°C. Before each injection, tamoxifen was first warmed in the Eppendorf tubes to 37°C in a water bath and then injected intraperitoneally.

### Tissue processing

Tissue was dissected and first fixed with 4% paraformaldehyde (Santa Cruz Biotechnology, CAS 30525-89-4) for 24–48 hours at 4°C, and then rinsed 3 times with PBS 5 minutes each, decalcified with 15%–30% EDTA decalcifying PBS solution until soft as judged by manual palpation (typically days to weeks at 4°C, depending on the age of the tissue), and then immersed in 30% (w/v) sucrose (Sigma-Aldrich, S0389) at room temperature for a few hours until the tissue dropped, or at 4°C overnight, and placed in OCT for cryosectioning. Frozen sections of 14 or 16 μm in thickness were collected serially, starting with the medial femur cartilage, and ending with the lateral edge of the joint.

### Cell proliferation assay

EdU (Invitrogen, A10044) was dissolved in PBS and given via intraperitoneal injection at a dose of 1.25 mg/mouse/day up to 5 days prior to sacrifice, as indicated above. A Click-iT Imaging Kit (Invitrogen, C10337) and Alexa Fluor 647 (Invitrogen A10277) were used for EdU detection in cryosections.

### Histomorphometry

Frozen sections were stained following protocols published online at the University of Rochester Center for Musculoskeletal Research (https://www.urmc.rochester.edu/musculoskeletal-research/core-services/histology/protocols.aspx).

### Immunofluorescent staining

#### Detection of aggrecan protein.

Tissue sections were first permeabilized with 0.5% Triton X-100 for 30 minutes to 1 hour at room temperature, followed by enzymatic permeabilization using hyaluronidase (Sigma-Aldrich, H3506) and blocked using serum from the species the secondary antibody was raised in. Anti-aggrecan antibody (Chemicon, AB1031; 1:200) was incubated with sections overnight at 4°C and followed by secondary antibody conjugated to Alexa Fluor 647 (Invitrogen, A-21245) at 1:200 for 30 minutes at room temperature. To ensure accurate protein signal detection, Alexa Fluor 647, a far-red dye with photon emissions primarily outside the human visible spectrum, was selected to eliminate the background of cartilage tissue autofluorescence.

#### Detection of tdTomato protein (pertaining to mRNA FISH combined with immunofluorescence).

Primary antibody (Rockland anti-RFP, 600-401-379; 1:250) was incubated for either 2 hours at room temperature or overnight at 4°C followed by secondary antibody staining with goat anti-rabbit (Alexa Fluor 488, Invitrogen, A-11034 or Alexa Fluor 594, Invitrogen, R37117) 1:200 for 30 minutes at room temperature. The selection of Alexa Fluor 488 or 594 was based on optimization assays to ensure sufficient signal-to-noise ratio of tdTomato protein compared with background or autofluorescence of cartilage tissue.

### mRNA FISH combined with immunofluorescent staining

Probes and reagents were purchased from Advanced Cellular Diagnostics (ACD, 323136 and 323110) following the manufacturer’s protocol. Cryosections (16 μm) were prepared per ACD’s protocol. Pepsin (Sigma-Aldrich, R2283) was used for antigen retrieval followed by mRNA probe hybridization (ACD, 439101 [*ACAN*]; ACD, 407221-C [*Col2*]; Opal dyes: Akoya Biosciences, Opal 520, FP1487001KT; Opal 650, FP1496001KT) and immunofluorescent staining to recover tdTomato protein digested by Pepsin, using the anti-RFP antibody staining protocol described in Immunofluorescent staining above. The fluorescence assays were designed based on selection of fluorescent dyes with distinct excitation and emission spectra per manufacturer database (Invitrogen, Akoya Biosciences) and https://www.fpbase.org and based on optimization assays to ensure a sufficient signal-to-noise ratio and spectral separation to comply with ACD and Leica confocal imaging guidelines.

### Image acquisition

Images were acquired with a Nikon fluorescence widefield microscope, Nikon Element confocal, Leica SpE or Sp8 confocal microscope (40× or 60× oil) as single images, *Z*-stacks, or tile scans. Scanning parameters were determined using manufacturer-recommended protocols. Individual *Z* optic sections acquired via laser confocal microscopes were analyzed as equivalent to tissue sections. For maximum intensity projection (projection of the brightest signals along the *z* axis), the *z*-axis thickness within 16 μm was selected to be smaller than 20 μm, the typical diameter of a chondrocyte, to avoid positional artifacts in the maximum intensity projection.

### Imaging processing

Images were acquired as individual fluorescent panels and merged using ImageJ (Fiji) open-source image-processing software. Individual panels may have been adjusted for brightness and contrast using Fiji, which was performed on the entire image during image processing. The original images may have been cropped to display the articular cartilage for adequate visualization. The Photoshop 2023 crop tool was used by filling in preset dimensions and/or preset resolution of at least 300 dpi. The Photoshop 2023 resizing function was also used to reduce image size to comply with the file size restriction required for journal submission.

### Quantification

#### Articular cartilage homeostasis.

Percentage of tdTomato^+^ articular chondrocytes was determined by calculating tdTomato^+^ among DAPI^+^ articular chondrocytes within the articular cartilage, excluding cells at the periphery of articular cartilage or transition tissues such as entheses or soft tissue attachment points. The boundary of the articular cartilage was defined by the tidemark.

#### SFZ chondrocytes in articular cartilage homeostasis.

Percentage tdTomato^+^ SFZ articular chondrocytes was calculated as flat tdTomato^+^ cells/DAPI^+^ SFZ chondrocytes. SFZ chondrocytes were defined as flat cells in the top 2 layers of articular chondrocytes, superficial to the underlying middle and deep zones, excluding the free cells outside the cartilage matrix, or tissue without 3-zonal architecture typical of articular cartilage (e.g., the periphery of articular cartilage or transition tissues such as entheses or soft tissue attachment points).

#### Articular cartilage wounds.

Percentage tdTomato^+^ wound cells was calculated as tdTomato^+^ cells/DAPI^+^ cells in wounds. The boundary of the wounds was determined based on brightfield, fluorescence images, or histology of adjacent sections, excluding superficial or surrounding soft tissue extensions outside the wound proper.

#### FISH data analysis.

Quantification of signals was performed using ImageJ software per guidelines for semiquantitative image analysis published by RNAscope/Advanced Cell Diagnostics (https://acdbio.com/).

### Statistics

Statistical analysis was performed with Prism 9 (GraphPad Software, Inc.). All *t* tests were 2-tailed. ANOVA is specified as 1- or 2-way in figures. A *P* value of less than 0.05 was considered significant and indicated accordingly in figures: *P < 0.05, **P < 0.01, ***P < 0.001, ****P < 0.0001. Results were based on quantification of at least 3 sections per group analyzed. Detailed description and output of all statistical tests are included in figure legends. All data are presented as mean ± SD.

### Study approval

The study was approved by the IACUC at Massachusetts General Hospital, Boston, Massachusetts, USA.

### Data availability

All relevant data are available in the manuscript, Supplemental Figures, Supporting Data Values, or from the corresponding author upon request.

## Author contributions

MM, HMK, and DTS conceived the study. MM designed and performed all experiments and acquired and analyzed the data. MM and JLM wrote the manuscript and generated figures. JLM contributed to data acquisition and analysis. The order of co–first authors was based on the relative overall contribution. HMK and DTS contributed to experimental design, data analysis, and critique of the manuscript. CRB contributed to experiments, data acquisition and analysis, and scholarly input. NB, TO, and MLS contributed to data presentation and scholarly input. MAR contributed to trouble shooting of the surgical approach and scholarly input. LSO and TJG provided scholarly input. DB contributed to the leptin receptor experiments and scholarly input. AW contributed to data acquisition relating to histochemistry and scholarly input. All authors critiqued and approved the manuscript.

## Supplementary Material

Supplemental data

Supporting data values

## Version 1 09/08/2023

Electronic publication

## Version 2 11/27/2023

Ninib Baryawno's name was corrected
