# Supplementary material for: Adult Prg4+ progenitors repair long-term articular cartilage wounds in vivo
Source: JCI Insight. 2023 Sep 8;8(17):e167858. doi: 10.1172/jci.insight.167858 (PMC10544199; doi:10.1172/jci.insight.167858)
Supplement: Supplemental data [file jciinsight-8-167858-s007.pdf]

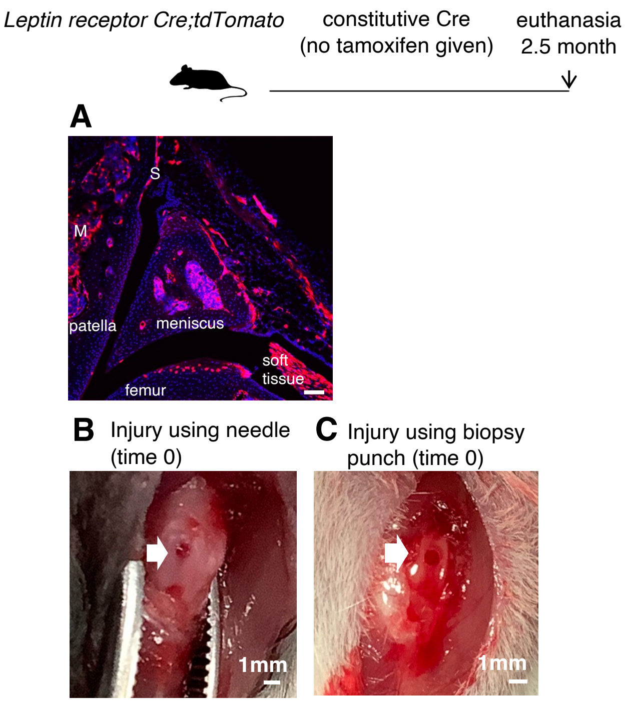

Supplemental figure 1 Diverse tissue labeling with constitutive (non-inducible) Leptin Receptor Cre, and injury models used in this study

A. homeostasis: fluorescent images of the knee of *Leptin Receptor Cre;rosatdTomato* mice, 2.5 months of age. S, synovium; M, bone marrow. Red: tdTomato. Blue: DAPI nuclei stain. Scale Bar: 50 µm.

B-C. mouse knee surgical wounds at time 0; (B), using needle; (C), using core biopsy punch. Arrow points to the surgical wound. Scale Bar: 1 mm.

Individual panels might be adjusted for brightness and contrast and displayed using Fiji. The original images may be cropped to display the articular cartilage for adequate visualization.

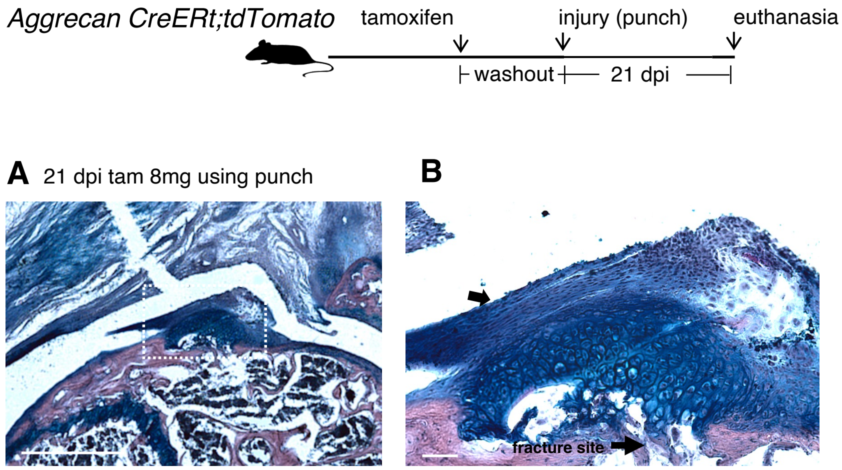

Supplemental figure 2 Wound architecture 21 dpi

A,B. Alcian Blue staining of a nearby section to that shown in figure 1G (given tamoxifen 8mg and using punch).

Arrow: superficial soft tissue surrounding the wound proper.

Long Arrows: site of subchondral bone fracture.

Patella tendon was cut during tissue processing to ensure penetration of tissue processing reagents.

Scale Bar: 500 µm in the uppermost panel (a), and 50 µm elsewhere.

Individual panels might be adjusted for brightness and contrast and displayed using Fiji. The original images may be cropped to display the articular cartilage for adequate visualization.

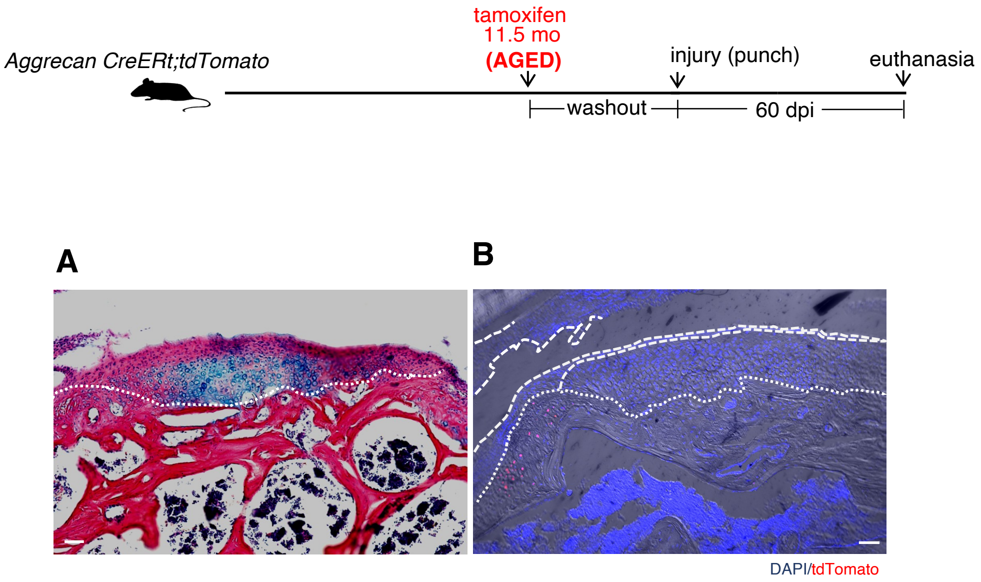

Supplemental figure 3 Wound architecture (aged mice), 60 dpi

A,B. Aged (11.5 months) AggrecanCreERt2;rosatdTomato, 60 dpi wound (punch); tamoxifen 4mg, 13 weeks washout to allow observation of any proliferation of aggrecan-expressing marrow stromal cells, and microfracture using 0.3mm biopsy punch (n=2).

(A): Alcian Blue.

(B): fluorescent confocal image of an adjacent section.

Dashed contours outline the superficial soft tissue surrounding the wound.

Dotted contours: the wound border.

Red: tdTomato. Blue: DAPI nuclei stain.

Scale Bar: 50 µm.

Individual panels might be adjusted for brightness and contrast and displayed using Fiji. The original images may be cropped to display the articular cartilage for adequate visualization.

A

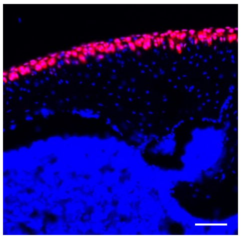

Supplemental figure 4

A. fluorescent images of *Prg4 creERt;rosa tdTomato* mouse knee following similar protocol as in figure 2A (homeostasis), given higher tamoxifen at 12mg.

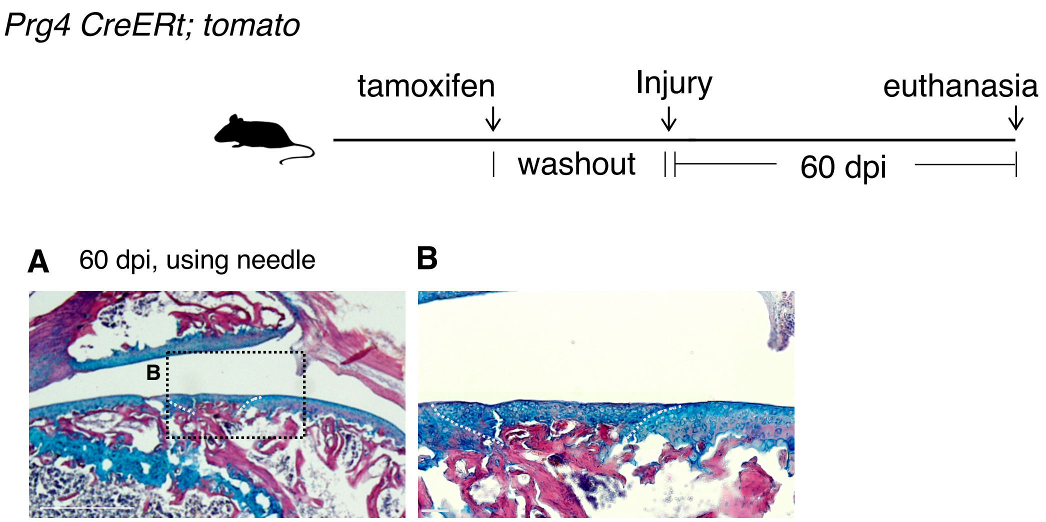

Supplemental figure 5 Wound architecture, 60 dpi

A,B. Alcian Blue stained nearby section to figure 2I, with mice injured using needle and euthanized at 60 dpi. Dotted contours: the wound border. Scale Bar: 500 µm (A), 50 µm (B). Individual panels might be adjusted for brightness and contrast and displayed using Fiji. The original images may be cropped to display the articular cartilage for adequate visualization.

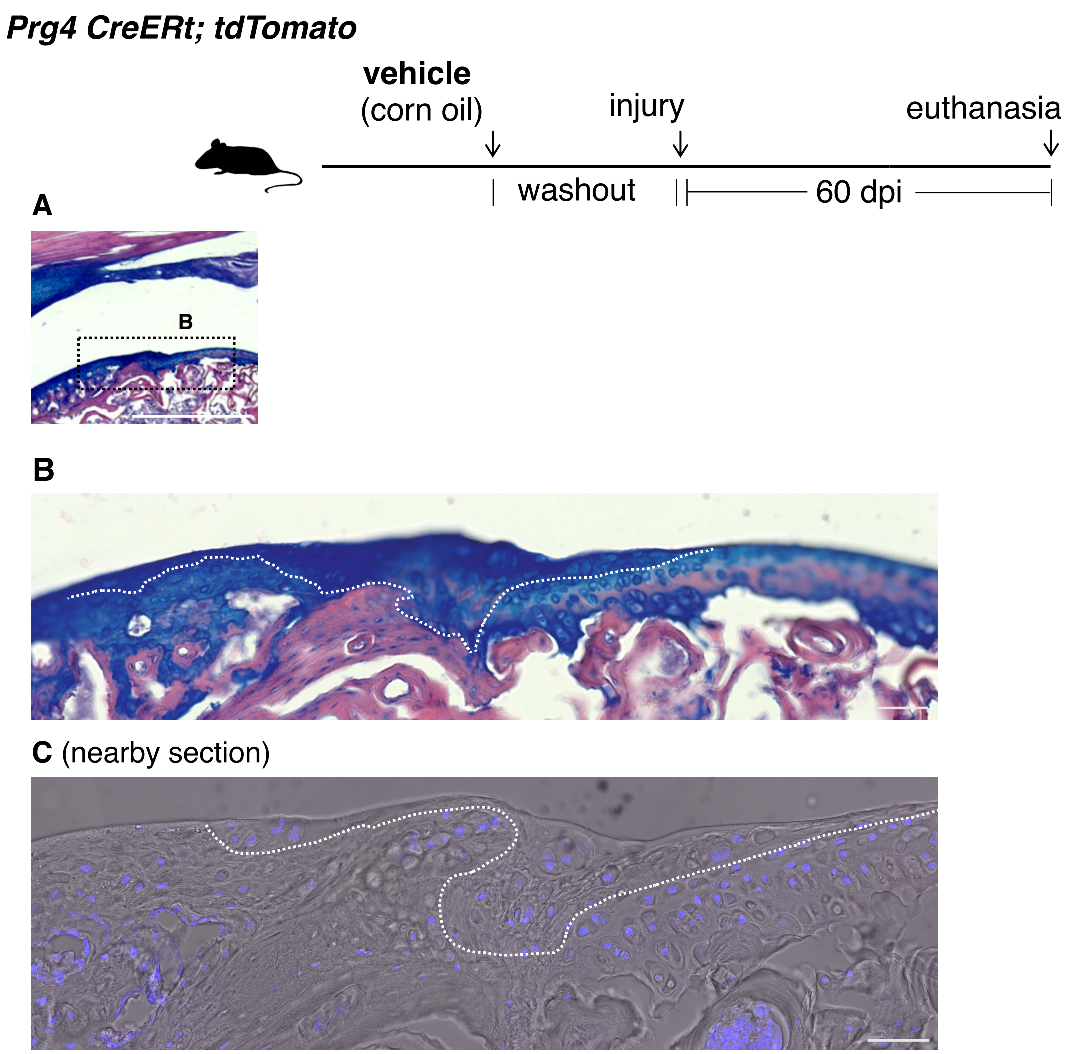

Supplemental figure 6 Vehicle control

A-C. Alcian blue (A,B) and fluorescent confocal (C) images of Prg4 creERt; rosa tdTomato knee wound (using punch), receiving only corn oil and euthanized at 60 dpi (n=2).

Dotted contours: the wound border. Scale Bar: 500 µm (A), 50 µm (B). Individual panels might be adjusted for brightness and contrast and displayed using Fiji. The original images may be cropped to display the articular cartilage for adequate visualization.

supplemental fig.7

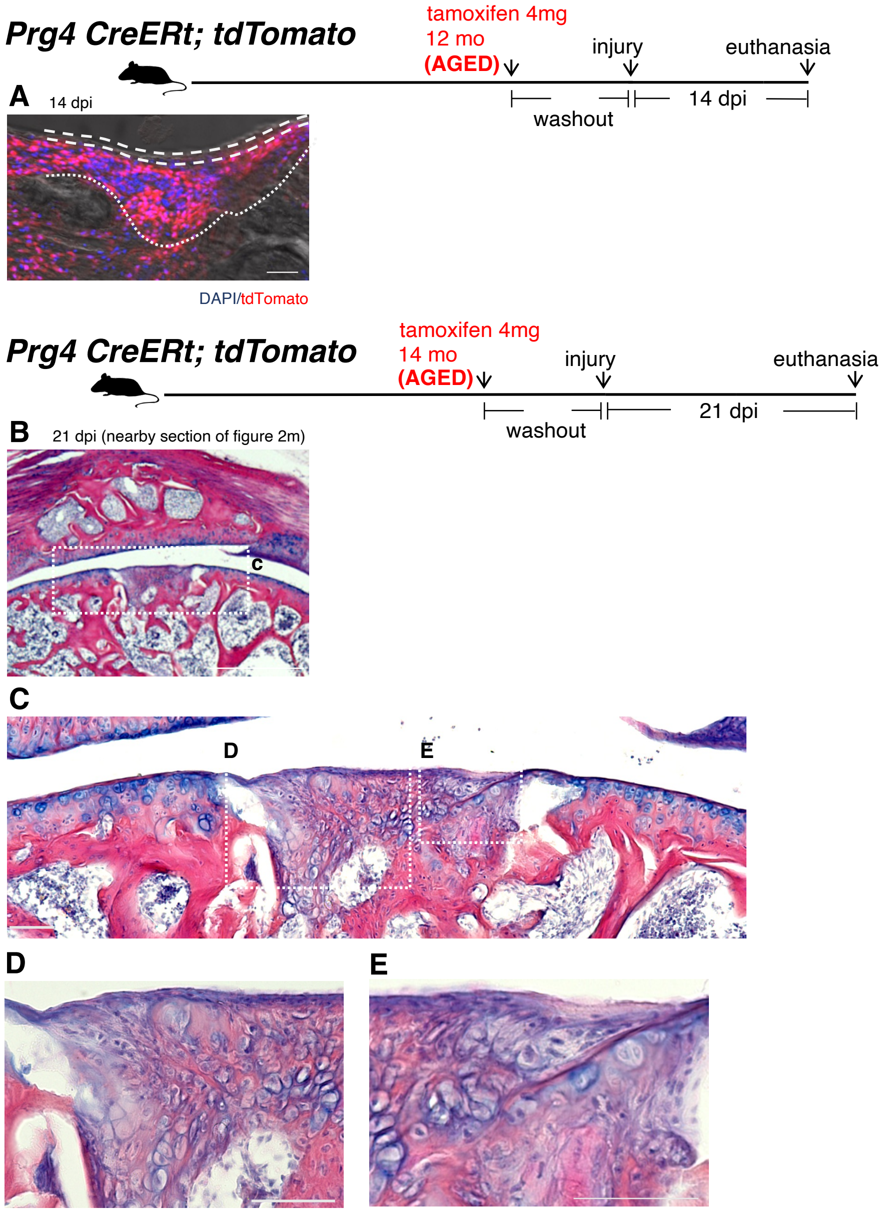

Supplemental figure 7: Wound architecture (aged mice)

A. 14 dpi: fluorescent image of *Prg4 creERt;tdTom* knee wound (needle), given 4mg tamoxifen, followed by washout, injury and euthanasia at 14 dpi .

Red: tdTomato. Blue: DAPI nuclei stain. Scale Bar: 50 µm.

B-E. 21 dpi: Alcian blue staining of Prg4 creERt;tdTom knee wound of a nearby section to that shown in figure 3A, given 4mg tamoxifen, followed by washout, injury and euthanasia at 21 dpi. Scale Bar: 500 µm (B), 50 µm elsewhere. Individual panels might be adjusted for brightness and contrast and displayed using Fiji. The original images may be cropped to display the articular cartilage for adequate visualization.

supplemental fig.8

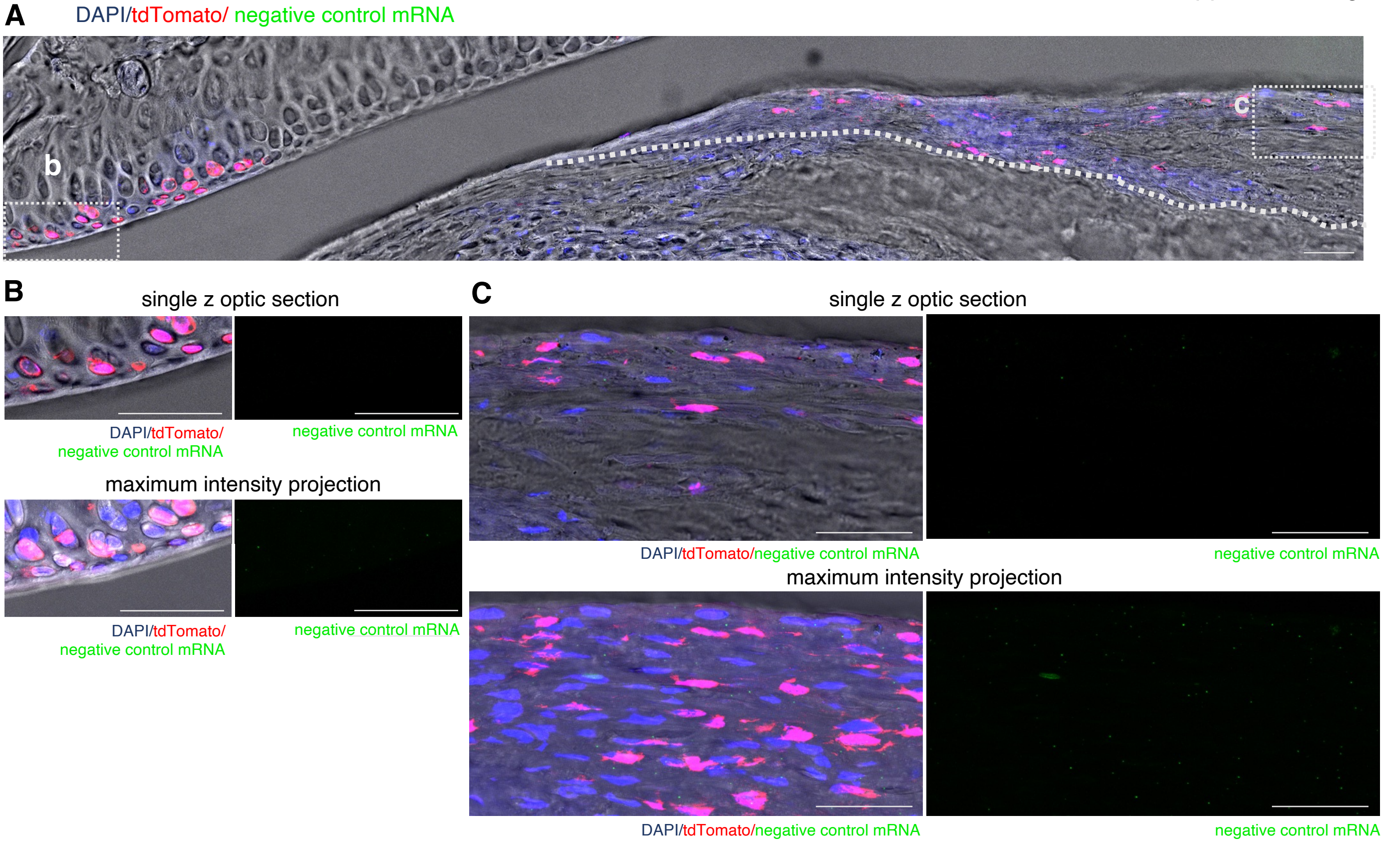

Supplemental figure 8 Aggrecan and Col2 mRNA in situ experimental controls related to figure 4

A-C: Aggrecan mRNA in situ hybridization negative controls using negative control mRNA probes and anti-RFP antibody.

(A): overview.

(B,C): high magnification insets.

Dotted contours: the wound border.

Blue: DAPI. Red: tdTomato. Red was pseudo-colored based on the staining signals of anti-tdTomato secondary antibody. Green: ACD negative control mRNA probe. Green was pseudo-colored based on the staining results of fluorescent mRNA in situ hybridization using far-red fluorophores to eliminate background associated with cartilage matrix auto-fluorescence.

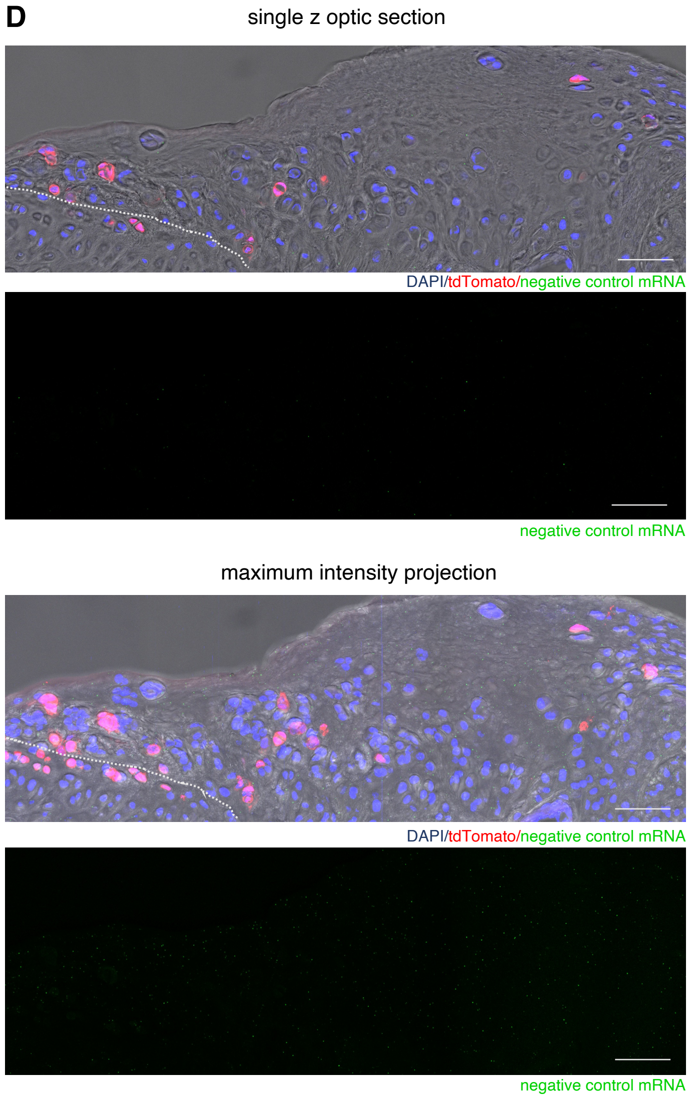

Supplemental figure 8 (continued,)

D: Col2 mRNA in situ hybridization negative controls, as in A-C.

Scale bars: 50 μm. Individual panels might be adjusted for brightness and contrast and displayed using Fiji. The original images may be cropped for adequate visualization.

Dotted contours: the wound border.

Blue: DAPI. Red: tdTomato. Red was pseudo-colored based on the staining signals of anti-tdTomato secondary antibody. Green: ACD negative control mRNA probe. Green was pseudo-colored based on the staining results of fluorescent mRNA in situ hybridization using far-red fluorophores to eliminate background associated with cartilage matrix auto-fluorescence.

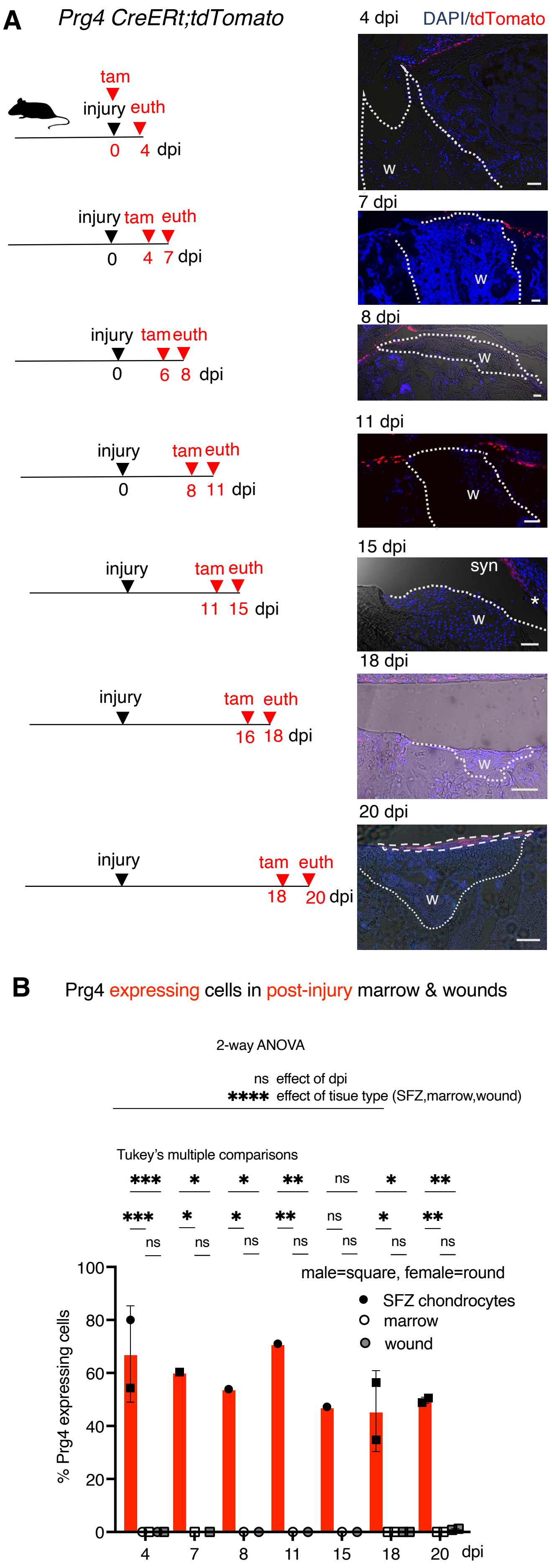

Supplemental figure 9 % Prg4 expressing cells in post-injury marrow and wounds

A. fluorescent images of Prg4 CreERt; rosa tdTomato with injury first then tamoxifen (reversing the sequence of tamoxifen and injury used in figure 2), to detect Prg4 expressing cells in post-injury marrow and wounds, showing post-injury marrow, wounds ("w"), articular chondrocytes, and synovium ("syn"). Tamoxifen 4 mg was given for all time points, except 6mg for 4,6,8,11 dpi with euthanasia within 2-4 days. Dashed contours outline the superficial soft tissue surrounding the wound. Dotted contours: the wound border. At 20 dpi, soft tissue surrounding the wound contained some tdTomato+ cells but these were not included in wound cell counts due to their locations outside the wound proper. Red: tdTomato. Blue: DAPI nuclei stain. Scale bars: 50 µm. Individual panels might be adjusted for brightness and contrast and displayed using Fiji. The original images may be cropped to display the articular cartilage for adequate visualization.

B. Quantitative summary: % Prg4 expressing (tdTomato) cells in tissues post-injury. (n=2 for groups given tamoxifen at 0, 16 or 18 dpi, and n=1 for the other groups). Two-way ANOVA: interaction, F=0.7812 (df between groups=12 and within groups=9), p=0.6622; time effect (wound dpi), F=0.7492 (df between groups=6 and within groups=9), p=0.6255; tissue effect (SFZ chondrocytes, marrow, and wound), F=150.2 (df between groups=2 and within groups=9), p=<0.0001; Tukey's multiple comparisons shown. All data were presented as mean ± s.d.

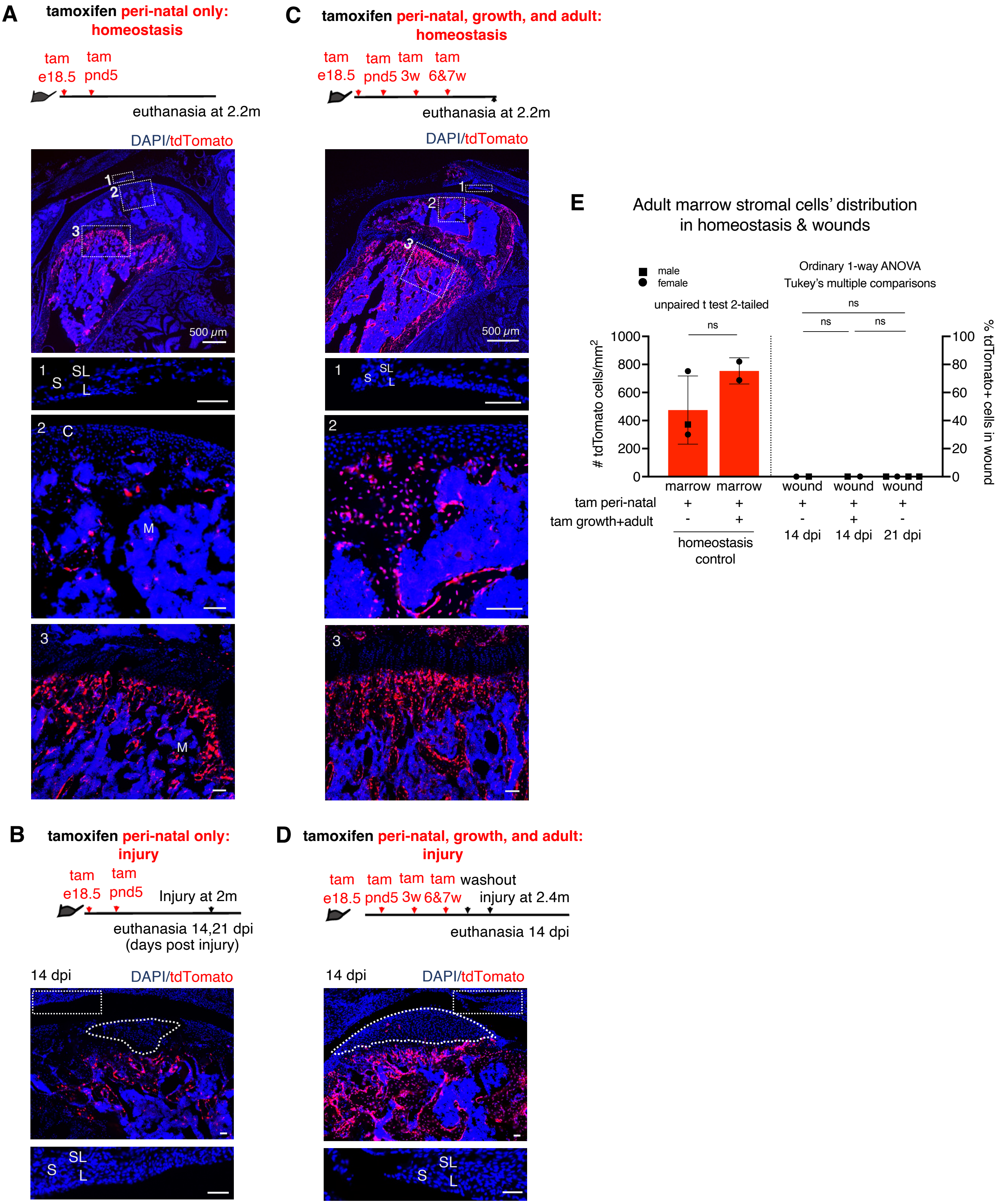
A
tamoxifen peri-natal only:
homeostasis
tam
e18.5
tam
pnd5
euthanasia at 2.2m
DAPI/tdTomato
1
2
3
500 μm
1 SL S L
2 C
M
3
M
B
tamoxifen peri-natal only:
injury
tam
e18.5
tam
pnd5
Injury at 2m
euthanasia 14,21 dpi
(days post injury)
14 dpi
DAPI/tdTomato
S SL L
C
tamoxifen peri-natal, growth, and adult:
homeostasis
tam e18.5
tam pnd5
tam 3w
tam 6&7w
euthanasia at 2.2m
DAPI/tdTomato
1
2
3
500 μm
1 S SL L
2
3
D
tamoxifen peri-natal, growth, and adult:
injury
tam e18.5
tam pnd5
tam 3w
tam 6&7w
washout
injury at 2.4m
euthanasia 14 dpi
14 dpi
DAPI/tdTomato
S SL L
E
Adult marrow stromal cells' distribution
in homeostasis & wounds
male
female
unpaired t test 2-tailed
ns
Ordinary 1-way ANOVA
Tukey's multiple comparisons
ns
ns
ns
tdTomato cells/mm2
% tdTomato+ cells in wound
1000
800
600
400
200
0
100
80
60
40
20
0
marrow marrow wound wound wound
tam peri-natal + + + + +
tam growth+adult - + - + -
homeostasis
control
14 dpi 14 dpi 21 dpi

Supplemental figure 10 Adult-marrow-dwelling stromal cells (peri-natal Sp7-lineage) are not major contributors to cartilage wound repair

A,B: Peri-natal tamoxifen labeling only.

(A). homeostasis: fluorescent images of knees of Sp7 (Osterix) creERt; rosa tdTomato mice showing tdTomato cells in adult bone marrow after receiving tamoxifen 0.1 mg/g during peri-natal time points (e18.5, n=1; e18.5+post-natal-day5, n=2) and euthanasia at 2.2m. High magnification views in insets (1-3). C: articular cartilage; M: marrow; S: synovium; L: lining; SL: synovium sublining.

(B). injury: mice received tamoxifen at peri-natal time points, followed by injury at 2m and euthanasia at 14 dpi (n=2). High magnification view of synovium in inset.

C,D: Peri- and post-natal tamoxifen labeling.

(C). homeostasis: fluorescent images of knee after receiving tamoxifen 0.1 mg/g at e18.5+post-natal-day5, 1mg at 3 weeks, 2mg at 6 weeks, and 2mg at 7 weeks of age (n=2), with euthanasia at 2.2m. High magnification views in insets (1-3). C: articular cartilage; M: marrow; S: synovium; L: lining; SL: synovium sublining.

(D). injury: mice followed the same tamoxifen protocol as in homeostasis (C) at e18.5, followed by 2 weeks washout after the last tamoxifen injection, injury at 2.4m, and euthanasia at 14 dpi (n=2) or 21 dpi (n=4). Dotted line outlines the cartilage wound.

Red: tdTomato. Blue: DAPI. Scale Bar: 50 µm unless otherwise indicated.

E. Quantitative summary: the abundance of tdTomato cells in adult marrow in homeostasis (left bars) and wounds (right bars). Sample size: as described above. For marrow homeostasis control, unpaired two-tailed student's t-test: t=1.489, df=3, p=0.2331; for wounds, one-way ANOVA: F = 4.974 (df between groups=2 and within groups=5 , p=0.0647; Tukey's multiple comparisons shown. All data were presented as mean ± s.d.

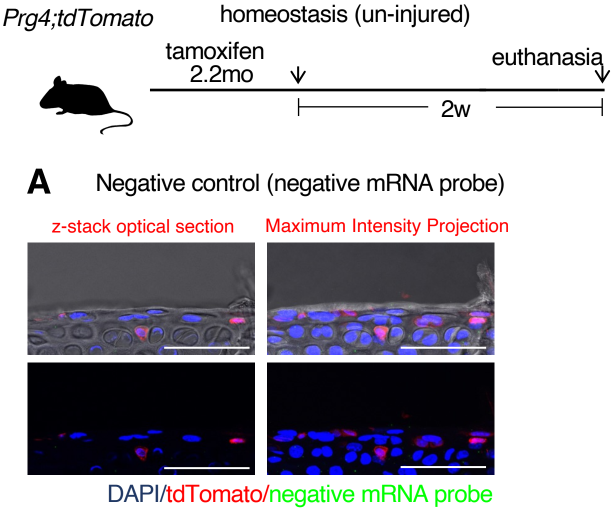

Supplemental figure 11

A: negative control in Aggrecan mRNA in situ hybridization assay: single z optic section vs. maximum intensity projection. Scale bars: 50 µm. Blue: DAPI. Red: tdTomato. Red was pseudo-colored based on the staining signals of anti-tdTomato secondary antibody. Green: ACD negative control mRNA probe. Green was pseudo-colored based on the staining results of fluorescent mRNA in situ hybridization using far-red fluorophores to eliminate background associated with cartilage matrix auto-fluorescence.
